# Fist sign-associated post ambulatory swollen hands

**DOI:** 10.1016/j.jdcr.2023.04.018

**Published:** 2023-04-29

**Authors:** Philip R. Cohen

**Affiliations:** a Department of Dermatology, University of California, Davis Medical Center, Sacramento, California; b Touro University California College of Osteopathic Medicine, Vallejo, California

**Keywords:** ambulatory, fist, hands, hiking, post, potash, running, sign, swollen, walking

## Introduction

A positive fist sign is characterized by the inability to make a fist.^1^ Post ambulatory swollen hands (POTASH) is an acquired condition that occurs in some individuals after running, hiking, or walking.^2^ The features of a man with post ambulatory hand swelling-associated positive fist sign is described.

## Case report

A healthy 64-year-old man presented with acute-onset positive fist sign of both hands; he had just completed a 13.1-mile race. For 3 hours and 52 minutes, he continuously ambulated; the ambient temperature was 70 degrees Fahrenheit. Similar to prior half marathons in which he had participated, the asymptomatic bilateral progressive swelling of his hands began after 1 hour of running.

**Fig 1:**
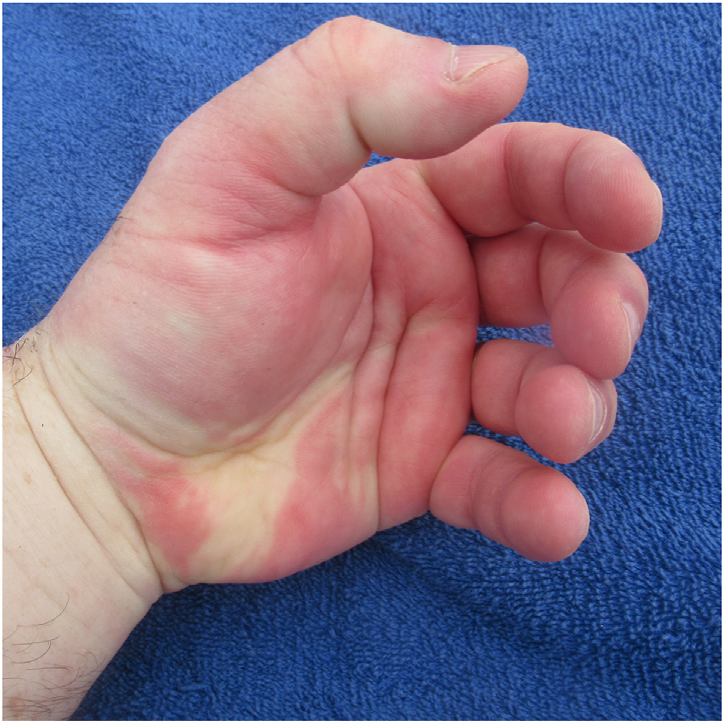
After completing a half marathon, a 64-year-old man had a positive fist sign. He could not clench the fingers on his left hand into the palm to make a fist.

**Fig 2:**
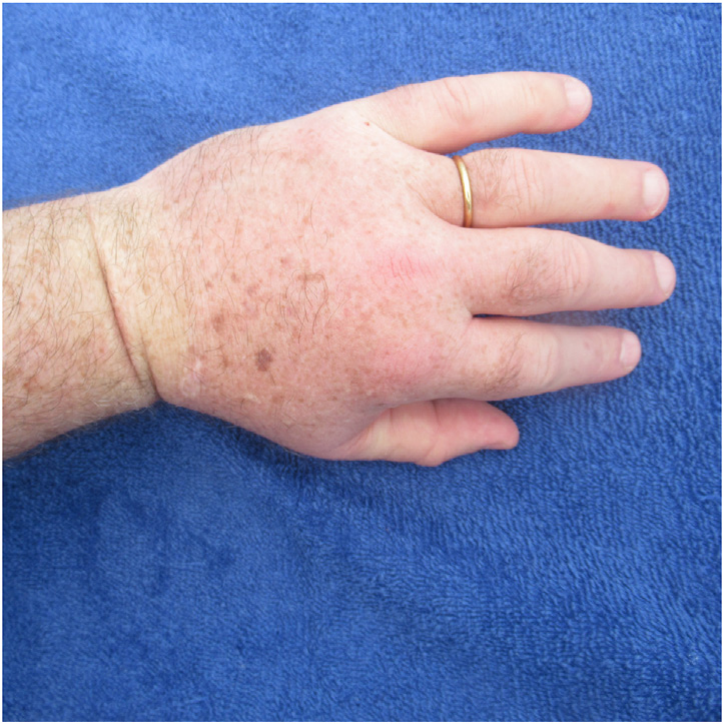
The man’s left hand and its digits are markedly swollen. The prominent swelling can readily be observed proximal and distal to the ring on his left fourth finger.

Examination demonstrated that he was not able to clench his fingers into his palm to make a fist; the effort resulted in erythema of the digits and palms (Fig 1). In addition, painless edematous hands, fingers, and thumbs of both upper extremities were observed; the swelling was readily noted proximal and distal to the ring on his left fourth finger (Fig 2). His dorsal hands and digits showed changes from chronic exposure to ultraviolet radiation; however, hives and angioedema, pruritus, and swelling of other body sites were absent.

**Fig 3:**
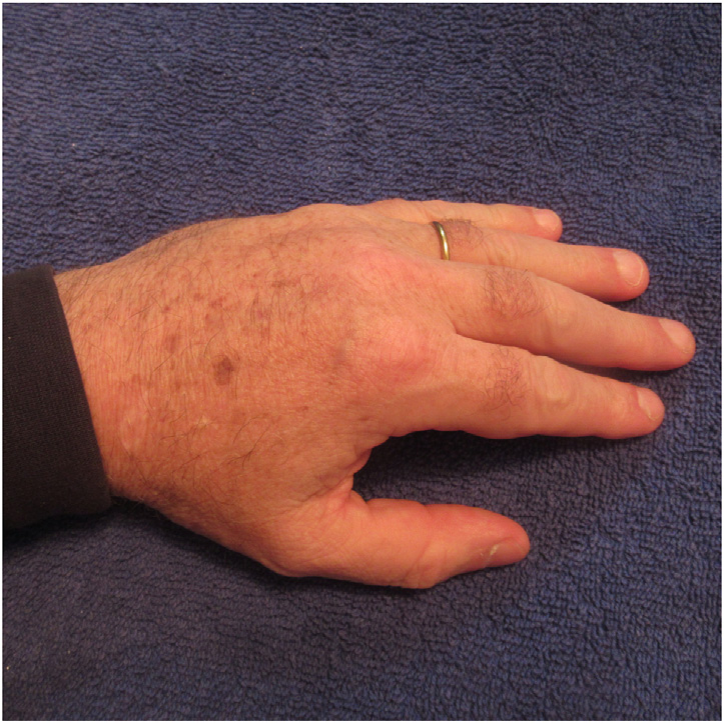
Spontaneous resolution of the man’s swollen hands and digits began approximately 1 hour after he stopped running; within 2 hours, his left hand and its digits were normal in appearance.

**Fig 4:**
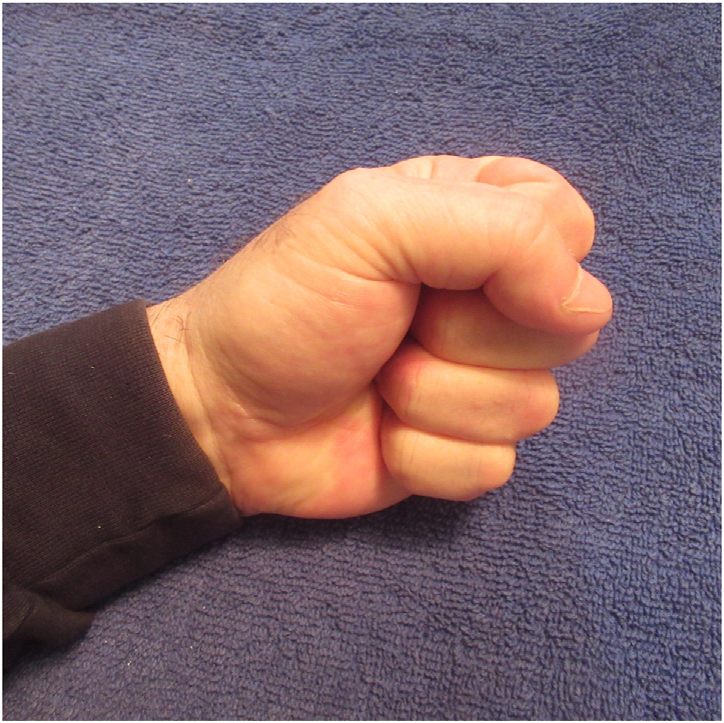
After the swelling resolved spontaneously, the man was able to make a fist by clenching his fingers into his palm. He now had a negative fist sign.

Correlation of the patient’s history and clinical presentation established the diagnosis of post ambulatory hand swelling (POTASH) with an associated positive fist sign. Similar to his previous episodes of post ambulatory swollen hands, the swelling of his hands and their digits eventually resolved spontaneously once he had finished the race and stopped running (Fig 3). Indeed, within 2 hours after he discontinued ambulating, he could clench his fingers to make a fist (Fig 4).

## Discussion

POTASH is characterized by recurrent, ambulation-induced, asymptomatic, bilateral acute swelling of the upper extremities distal to the wrists. Although the swelling is postulated to be caused by edema, the pathogenesis of this condition remains to be established. The disorder usually resolves spontaneously after ambulation is stopped.^2^, ^3^, ^4^, ^5^

This condition has been observed in runners, hikers, and walkers.^4^ It was originally described in the medical literature as post ambulatory hand swelling (PAHS) or big hand syndrome.^3^ Subsequently, it has been designated as post ambulatory swollen hands and referred to by the acronym POTASH.^2^^,^^5^ The features of previously reported individuals with swollen hands following ambulation are summarized in Table I.^2^, ^3^, ^4^, ^5^

**Table I:** Characteristics of individuals with swollen hands following ambulation

Author	Condition name	Causative factor	Positive fist sign	Comments	Ref
Ravaglia et al	PAHS∗	Walking†	Yes‡	A study of 1009 individuals who participated in a once monthly walking program in local parks.	^3^
Des Marais	Swollen hands	Hiking Walking	NS§	An article (published in a hiking and fishing journal) that reviewed possible etiologies and preventions for swollen hands when hiking or walking.	^4^
Cohen	POTASH‖	Running	Yes	Case reports of recurrent episodes of swollen hands in a sexagenarian that begin while running a half marathon and temporarily persist before spontaneously resolving after he completes the 13.1 miles.	^2^^,^^5^ ^CR^

*CR*, Current report; *NS*, not stated; *PAHS*, post ambulatory hand swelling; *POTASH*, post ambulatory swollen hands; *Ref*, references.

∗ The authors also referred to the condition as big hand syndrome.

† Swollen hands after walking was statistically observed more often in younger individuals as compared to older persons and dog owners as compared to nonowners.

‡ The assessment of hand swelling included difficulty to: (1) make a fist, or (2) remove rings, or (3) remove a watch or wrist band.

§ A description of the fingers of hikers and walkers starting to swell like sausages while they were ambulating on the trail was provided; however, the author did not discuss the fist sign in his article.

‖ The acronym POTASH is derived from post ambulatory swollen hands. POT are the first, second, and fourth letters of the word post. A, S, and H are the first letters of the words ambulatory, swollen, and hands.

The differential diagnosis of POTASH includes urticaria, conditions associated with fluid overload, and cellulitis. The absence of hives or angioedema in this patient excluded urticaria or an allergic reaction. Since the swelling was only localized to the distal hands without any edema of the legs or abdomen, accumulation of fluid from heart, kidney, or liver disease was also excluded. In addition, cellulitis was excluded because there were neither systemic symptoms (such as fever) nor local signs (such as warmth, tenderness, or persistent erythema) of infection. This patient’s rapid occurrence of bilateral swelling only limited to the hands--with an associated positive fist sign--was elicited by his participation in an activity in which there was prolonged running; therefore, he had POTASH.^2^, ^3^, ^4^, ^5^

A positive fist sign is a pathognomonic clinical feature of post ambulatory swollen hands.^2^^,^^3^^,^^5^ The affected individual is not able to tightly clench their fingers into the palm to form a fist. In addition to POTASH, some of the other potential etiologies for a positive fist sign include compartment syndrome, congenital conditions (such as Blau syndrome), endocrine disorders (such as acromegaly), giant lipoma (with associated carpal tunnel syndrome), intravenous drug use (with puffy hand syndrome), and rheumatologic diseases (such as remitting seronegative symmetrical synovitis with pitting edema).^1^^,^^6^, ^7^, ^8^, ^9^, ^10^

In patients with POTASH, the fist sign occurs secondary to edema.^2^, ^3^, ^4^, ^5^ Other causes of a positive fist sign include fibrosis, hemorrhage, infection, lymphatic obstruction, overgrowth of bone and soft tissue, space occupying lesion with nerve compression, and trauma.^1^^,^^6^, ^7^, ^8^, ^9^, ^10^ Depending on the etiology, the inability to make a fist will persist or may resolve either spontaneously, after corticosteroid treatment, or post-surgery.^1^, ^2^, ^3^, ^4^, ^5^, ^6^, ^7^, ^8^, ^9^, ^10^

In summary, POTASH is a benign condition that may occur in people who ambulate. The asymptomatic swelling of the hands and their digits typically prevents the individual from clenching their fingers into the palm; hence, a positive fist sign is a characteristic feature of POTASH. Although the etiology of POTASH is unknown, the condition is acquired and recurrent; spontaneous resolution typically occurs after the ambulatory activity has been discontinued.

## Conflicts of interest

None disclosed.
